# Survival outcome of local vs. radical excision in rectal gastrointestinal stromal tumor: a SEER database analysis

**DOI:** 10.1186/s12893-022-01485-3

**Published:** 2022-01-22

**Authors:** Jianchang Wei, Junbin Zhong, Zhuanpeng Chen, Qing Huang, Fang Wei, Qiang Wang, Jie Cao

**Affiliations:** 1 The First Affiliated Hospital, Jinan University, Guangzhou, China; 2 Department of General Surgery, Guangzhou Digestive Disease Center, Guangzhou First People’s Hospital, The Second Affiliated Hospital of South China University of Technology, Guangzhou, China

**Keywords:** Rectal gastrointestinal stromal tumor, Local excision, Radical excision

## Abstract

**Background:**

The choice of surgical strategy for patients with rectal gastrointestinal stromal tumor (GIST) remains controversial. This study aims to address whether the surgical procedure [local excision (LE) vs. radical excision (RE)] influences the survival outcomes.

**Methods:**

The information of the patients recruited in this study was obtained from the Surveillance, Epidemiology, and End Results (SEER) database. A survival curve was used to evaluate the differences in cancer-specific survival (CSS).

**Results:**

No significant difference was detected in the CSS between the LE and RE groups. Also, no significant differences were observed in the CSS between the two groups with respect to different T classification, N classification, tumor differentiation, tumor size, regional LN surgery, age, gender, race, chemotherapy, and radiotherapy. The T classification and age were independent prognostic factors in rectal GIST patients.

**Conclusions:**

LE and RE have similar survival time after surgery, and LE could be considered as an effective surgical approach for rectal GIST.

## Background

Gastrointestinal stromal tumor (GIST) is the most common mesenchymal tumor in the digestive tract [1]. Rectal GIST is rare, accounting for about 5% of all GISTs [2], but the malignancy of GIST in the rectum is higher than that at other sites and related to poor prognosis [3, 4].

Surgery is the crucial therapy for GIST, and the main goals of surgery of rectal GIST are to obtain negative resection margins and preserve the anal sphincter [5]. Currently, local excision (LE) and radical excision (RE) are feasible for rectal GIST, and the selected surgical approach is mostly the subjective opinion of surgeons [6]. Historically, rectal GIST is treated with RE, including abdominoperineal excision and total pelvic exenteration, as RE is associated with low local recurrence [7]. However, RE also causes large trauma and severe bowel dysfunction, which might be related to the decline in anorectal function due to anastomosis and impaired life quality because of stoma [7]. Lymph node (LN) metastasis is rare in GIST, and therefore, regional LN dissection is unnecessary [8, 9], deeming LE as a reasonable approach with minimal invasion to preserve the function of the anal sphincter [10], especially in the modern era of imatinib target therapy [10–12]. Recent studies have demonstrated that local recurrence does not differ between the two surgical approaches [13, 14]. LE also shows a prognosis similar to RE [6, 7]. Shu et al. found that patients who underwent LE had a prolonged overall survival, but the RE patients exhibited pronounced malignancy [13].

Nonetheless, the optimal surgical approach for rectal GIST is yet controversial due to the low disease incidence and the lack of evidence for large-scale population studies [6, 14–19]. In this retrospective study, we analyzed 154 rectal GIST patients in the public large population-based Surveillance, Epidemiology, and End Results (SEER) database and compared the long-term cancer special survival (CSS) outcomes of the two surgical treatments in order to define the optimal surgical strategy for rectal GIST.

## Methods

### Patient selection

Patients with rectal GIST from 1973 to 2015 were collected from the SEER database using SEER ∗ Stat 8.3.8 (http://seer.cancer.gov). Ethical consent was not required in this study as the patients’ information from the database is anonymous [20, 21].

The clinicopathological data of rectal GIST patients, including age, gender, race, differentiation, tumor, node, and metastasis (TNM) stage, surgical resection, tumor size, radiation, chemotherapy, and survival months were collected from the SEER database.

The primary tumor site was defined by the International Classification of Diseases for Oncology (ICDO) code: C20.9-Rectum. The tumor type of GIST was defined by the ICDO code: 8936/3: Gastrointestinal stromal sarcoma [20].

Patients with distant metastasis (M1), without complete clinical data of interest, without complete therapy information, failed follow-up, and undergone local tumor destruction were excluded from this analysis.

In order to distinguish different surgical methods, the RX Summ-Surg Prim Site (1998+) codes were restricted to “26, 27, 30–80.” We divided the surgery procedures into two groups: LE and RE. The LE group included patients treated with polypectomy or excisional biopsy with the pathological specimen (surgery encode 26, 27), while the RE group included patients treated with partial, subtotal, or total proctectomy, anterior resection, Hartmann’s operation, rectosigmoidectomy, and total proctectomy, including abdominoperineal resection, anterior/posterior resection, Miles’ operation, and Rankin’s operation (Surgery encode 30–80) [22]. Patients undergoing local tumor destruction (photodynamic therapy, electrocautery, cryosurgery, laser ablation and excision, curette, and fulguration) were excluded [22]. In addition, patients breaching the above inclusion criteria were excluded from the present study.

CSS is defined as the survival time from a patient’s diagnosis of the disease to death specific attributable to cancer.

### Statistical analysis

A Chi-square test was conducted to evaluate the difference in clinical characteristics between LE and RE groups. Kaplan–Meier survival curves and log-rank tests were conducted to evaluate the differences of CSS time between LE and RE groups. Univariate and multivariate Cox models were applied to determine hazard ratios (HRs) and 95% confidence intervals (CIs). All analyses were conducted using Empower (R) (www.empowerstats.com, X&Y solutions, Inc., Boston, MA, USA) and R (http://www.R-project.org).

## Results

### Patient characteristics

**Fig. 1:**
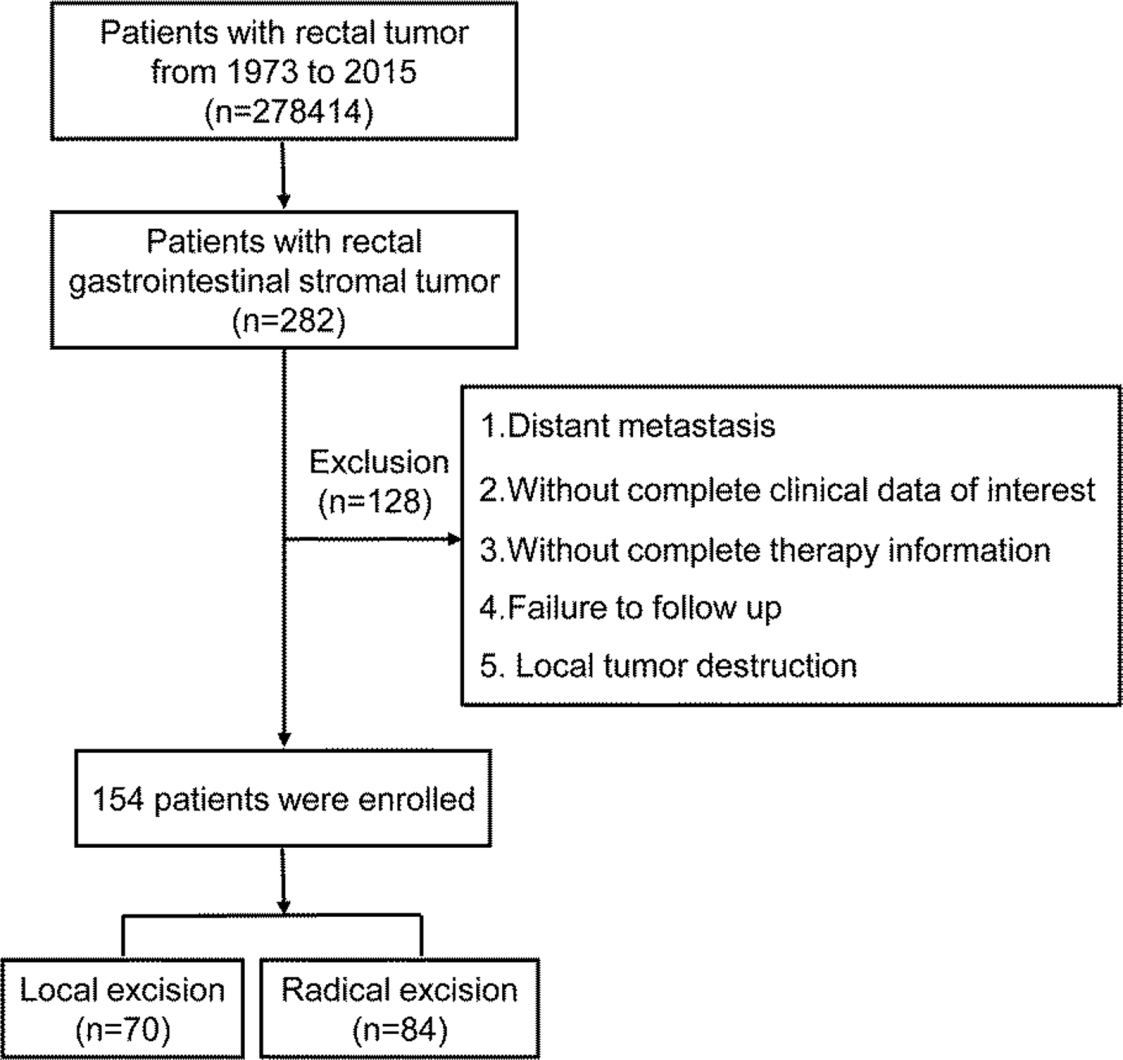
Flowchart of the patients’ selection in SEER database

The patient selection flow in this study is shown in Fig. 1. A total of 154 patients with rectal GIST were obtained from SEER database from 1973–2015; 70 (45.5%) underwent LE surgery and 84 (54.5%) underwent RE. All the patients enrolled finally in this study were diagnosed after the year of 2000, which indicated they were diagnosed and treated in the modern era of imatinib target therapy and they were comparable.

The clinicopathological characteristics of the two groups are summarized in Table 1. Overall, no significant associations were observed between age, gender, race, differentiation, tumor size, chemotherapy, and radiotherapy. T classification (p < 0.001), N classification (p = 0.002), and regional LN surgery (p < 0.001) differed significantly, i.e., patients in the RE group presented deeper tumor invasion, more LN metastasis, and more regional LN surgery than the LE group.

**Table 1:** Comparison of clinicopathological features between local and radical excision

Characteristics	Local excision n = 70, n (%)	Radical excision n = 84, n (%)	p
Age, years			0.056
≤ 60	30 (42.9%)	49 (58.3%)	
> 60	40 (57.1%)	35 (41.7%)	
Gender			0.744
Male	41 (58.6%)	47 (56.0%)	
Female	29 (41.4%)	37 (44.0%)	
Race			0.923
White	41 (58.6%)	49 (58.3%)	
Black	11 (15.7%)	15 (17.9%)	
Other	18 (25.7%)	20 (23.8%)	
Differentiation			0.603
Well/moderately	16 (22.9%)	18 (21.4%)	
Poorly/undifferentiated	7 (10.0%)	13 (15.5%)	
Unknown	47 (67.1%)	53 (63.1%)	
T classification			< 0.001
T1–2	27 (38.6%)	7 (8.3%)	
T3–4	6 (8.6%)	16 (19.0%)	
Unknown	37 (52.9%)	61 (72.6%)	
N classification			0.002
N0	35 (50.0%)	22 (26.2%)	
N+/unknown	35 (50.0%)	62 (73.8%)	
Tumor size, cm			0.222
≤ 3	41 (58.6%)	40 (47.6%)	
> 3	10 (14.3%)	10 (11.9%)	
Unknown	19 (27.1%)	34 (40.5%)	
Regional LN surgery			< 0.001
No	53 (75.7%)	15 (17.9%)	
Yes	3 (4.3%)	43 (51.2%)	
Unknown	14 (20.0%)	26 (31.0%)	
Chemotherapy			0.702
No	38 (54.3%)	43 (51.2%)	
Yes	32 (45.7%)	41 (48.8%)	
Radiotherapy			0.816
No	52 (74.3%)	61 (72.6%)	
Yes	18 (25.7%)	23 (27.4%)	

*LN* lymph node

### Survival analysis by clinicopathological characteristics

**Fig. 2:**
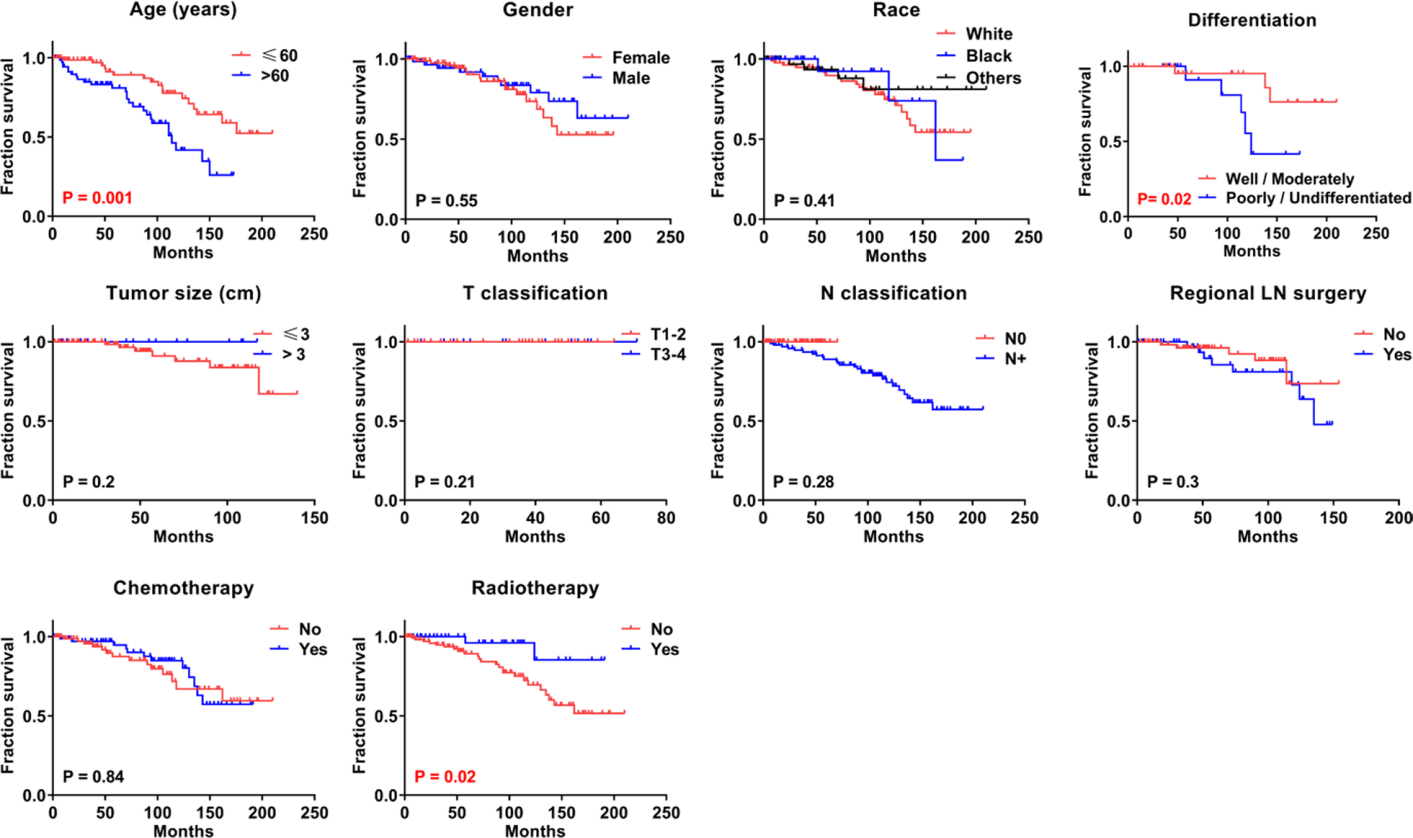
Cancer specific survival (CSS) analysis by clinicopathological characteristics of rectal gastrointestinal stromal tumor patients

The CSS of patients with rectal GIST was compared with respect to the clinicopathological characteristics. Significant differences were detected in age (p = 0.001), differentiation (p = 0.02), and radiotherapy (p = 0.02) (Fig. 2) in CSS for rectal GIST patients, while no significant differences were observed in CSS with respect to gender, race, tumor size, T classification, N classification, regional LN surgery, and chemotherapy (Fig. 2).

### Survival analysis by surgical approach

**Fig. 3:**
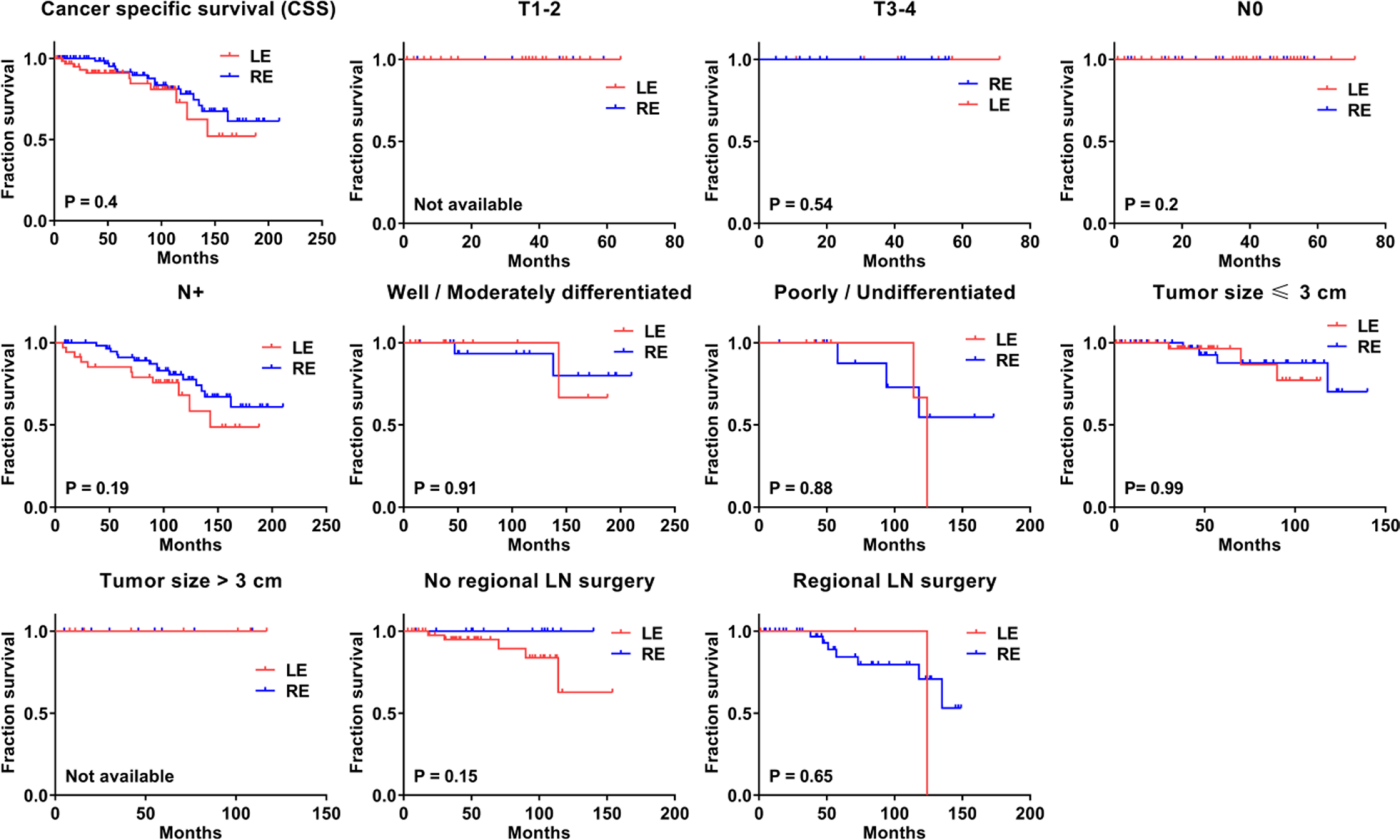
Cancer specific survival (CSS) analysis by local excision (LE) and radical excision (RE) in different pathological characteristics of rectal gastrointestinal stromal tumor patients

To investigate whether rectal GIST patients obtained a survival benefit from the surgical approach, we compared the CSS time between LE and RE groups. The median CSS in the LE group was 53 months, whereas that in the RE group was 46 months; however, no significant difference was observed between the two groups (log-rank = 0.704, p = 0.401) (Fig. 3).

**Fig. 4:**
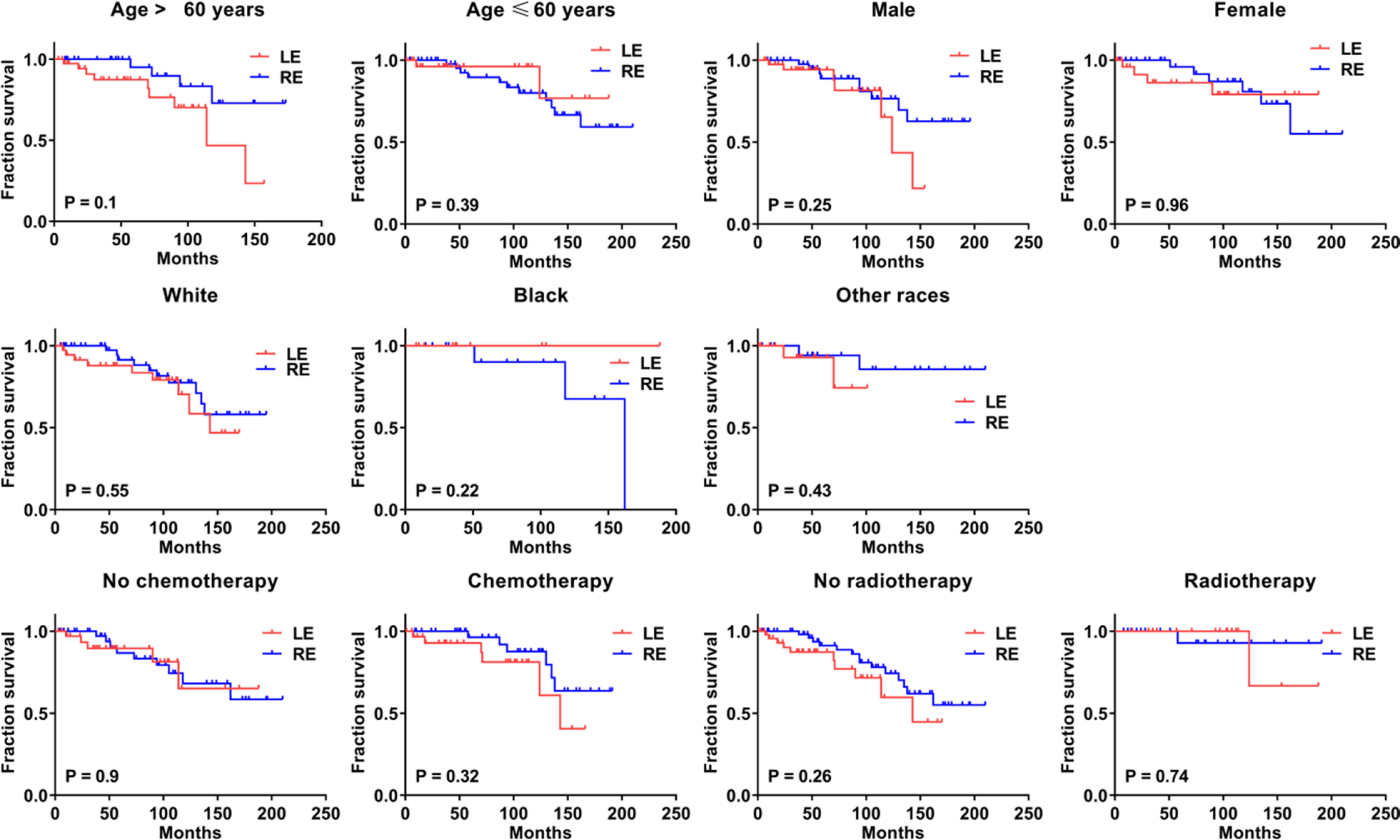
Cancer specific survival (CSS) analysis by local excision (LE) and radical excision (RE) in different clinic characteristics of rectal gastrointestinal stromal tumor patients

To further determine whether LE surgery effectuated the CSS time similar to that of the RE with respect to various clinicopathological characteristics, we compared the CSS curves. As shown in Figs. 3 and 4, no significant differences were detected in the CSS time between the two groups with respect to different T classification, N classification, tumor differentiation, tumor size, regional LN surgery, age, gender, race, chemotherapy, and radiotherapy (all p > 0.05).

### Subgroup survival analysis

To identify the independent prognostic factors for rectal GIST patients, we conducted univariate and multivariate Cox regression analysis. The T classification (p = 0.024) was an independent prognostic factor in patients with rectal GIST (Table 2), indicating that rectal GIST patients with T3–4 were at 1.7-fold risk of death as compared to those with T1–2. Interestingly, age was also identified as an independent prognostic factor, showing that patients aged > 60 years were at 2.6-fold risk of death as compared to those ≤ 60 years (p = 0.003). Conversely, race, differentiation, tumor size, N classification, regional LN surgery, chemotherapy, and radiotherapy were not independent prognostic factors for rectal GIST patients.

**Table 2:** Univariate and multivariate analysis for the rectum GIST patients

Characteristics	Univariate analysis			Multivariate analysis		
	HR	95% CI	p*	HR	95% CI	p**
Age						
≤ 60	1			1		
> 60	2.9	1.5–5.4	0.001	2.6	1.4–5.0	0.003
Race						
White	1					
Black	0.8	0.4–2.0	0.704			
Other	0.8	0.4–1.6	0.517			
Differentiation						
Well/moderately	1			1		
Poorly/undifferentiated	3.5	1.3–9.8	0.017	2.4	0.8–6.8	0.106
Unknown	2.1	0.9–5.2	0.094	1.8	0.7–4.4	0.199
Tumor size, cm						
≤ 3	1					
> 3	0.3	0.0–2.4	0.247			
Unknown	1.2	0.6–2.4	0.608			
T classification						
T1–2	1			1		
T3–4	1.2	1.1–6.2	0.043	1.7	1.3–2.9	0.024
Unknown	2.7	0.9–3.2	0.223	1.3	0.5–2.1	0.132
N classification						
N0	1					
N+/unknown	2.8	0.6–12.2	0.176			
Regional LN surgery						
None	1					
Yes	1.8	0.8–4.1	0.193			
Unknown	1.6	0.7–3.7	0.237			
Chemotherapy						
None	1					
Yes	0.8	0.5–1.1	0.170			
Radiotherapy						
None	1					
Yes	0.7	0.3–1.1	0.184			

*95% CI* 95% confidence interval, *HR* hazard ratio, *LN* lymph node

*p < 0.1 was considered significant in univariate Cox-Regression analysis

**p < 0.05 was considered significant in multivariate Cox-Regression analysis

## Discussion

Surgery is the leading therapy for GIST [5]. Currently, LE and RE are feasible surgical strategies for rectal GIST [6]. RE is associated with low local recurrence [7], but always results in large trauma, severe bowel dysfunction, and poor quality of life [7]. LE is a minimal invasion method to preserve the function of anal sphincter [10], especially in the modern era of imatinib target therapy [11]. Typically, LE is related to higher local recurrence and shorter survival time than RE.

Yasui et al. collected rectal GIST patients across 40 institutions from 2003 to 2007; however, only 24 cases were enrolled in the study due to the low incidence of this disease [12]. The study found that the local recurrence rate was 30.4% after curative resection, but that did not differ after LE (33.3%) vs. extended resection (28.6%) [12]. Shu et al. analyzed 71 rectal GIST patients from 2004 to 2017, including 42 patients who underwent LE and 29 patients who underwent RE, in a retrospective study. The study also showed that the two surgical approaches did not have any significant impact on recurrence-free survival [13]. Interestingly, the patients who underwent LE have longer overall survival than RE, but the RE patients were more moderate-high risk malignancy cases than those undergoing LE [13]. In addition, LE is a preferred surgery for rectal GIST with less injury and short hospital stay [13]. Guo et al. also found that LE has a similar clinical prognosis with RE, and LE can achieve short operative time, less operative bleeding, and a quick recovery, especially when combined with neoadjuvant therapy of imatinib [6, 7]. However, the optimal surgical strategy for rectal GIST remains controversial due to the low incidence and limited patient scale [6, 14–19].

In this retrospective study, we analyzed 154 rectal GIST patients in the SEER database. Although the number of patients enrolled was also limited, to the best of our knowledge, this is the largest population-based study on rectal GIST.

We found significant differences in CSS time for rectal GIST patients with respect to age, tumor differentiation, and radiotherapy, indicating that rectal GIST patients aged ≤ 60 years, with well/moderate differentiation and undergoing radiotherapy, exhibit a prolonged CSS time. Historically, GIST is considered radiation-resistant, and radiotherapy is not the predominant treatment for GIST. However, recent studies showed that radiation might be beneficial in advanced-stage GIST [23, 24]. The current results showed that radiotherapy benefits rectal GIST patients with a better prognosis, implying that radiotherapy may be a promising and potential treatment for rectal GIST but needs further investigation. Intriguingly, no significant differences were observed in the CSS time with respect to gender, race, tumor size, T classification, N classification, and regional LN surgery. The results further confirmed that regional LN surgery is unnecessary [9]. Imatinib is used as the first-line treatment for rectal GIST for a satisfactory oncological outcome [11]. Also, no significant differences were noted in the CSS time of chemotherapy in this study, but the detailed information of chemotherapy was unavailable, which we thought greatly influenced the result.

The present study aimed to compare the long-term survival of rectal GIST patients who underwent either LE or RE. Our results did not detect any significant difference in the CSS time between the two surgeries with respect to age, gender, race, tumor differentiation, tumor size, T classification, N classification, regional LN surgery, chemotherapy, and radiotherapy. These results suggested that the two surgical procedures are similar in terms of survival outcomes and a limited excision range, i.e., LE might be sufficient for rectal GIST patients.

Although we analyzed a large number of rectal GIST patients in the public SEER database, the present study has certain limitations. First, it is a retrospective study, and thus, bias is inevitable. Second, the lack of information on the neoadjuvant or adjuvant therapy of imatinib, distance of the tumor from anal verge and mitotic figures of the tumor, which might influence the survival of patients. Third, the lack of information on postoperative recurrence and the quality of life of rectal GIST patients might affect the choice of surgical strategy of surgeons. Thus, high-quality randomized controlled trials (RCTs) are imperative to elucidate the significance of both surgical approaches. Nonetheless, future studies would focus on the postoperative life quality of patients to determine the optimal approach for rectal GIST.

## Conclusion

Although RE achieves a prolonged surgical margin, no differences were detected in the CSS time. RE and LE have similar survival time after surgery. RE was not necessary for rectal GIST patients, and LE could be considered as an effective surgical approach for rectal GIST.

## Data Availability

The data used and analyzed during the study are available in SEER database (http://seer.cancer.gov), which is a public dataset online.

## Abbreviations

GIST: Gastrointestinal stromal tumor
LE: Local excision
RE: Radical excision
SEER: Surveillance, Epidemiology, and End Results
CSS: Cancer-specific survival
LN: Lymph node
HR: Hazard ratio
CI: Confidence interval
